# #TobaccoNoMore: Insights From Malaysia's Smoking Patterns and Their Implications for the Philippines

**DOI:** 10.1002/hsr2.70567

**Published:** 2025-03-11

**Authors:** John Patrick C. Toledo

**Affiliations:** ^1^ Department of Theology and Religious Education De La Salle University Manila Philippines

Dear Editor,

I've read with great interest the study entitled Exploring Prevalence and Sociodemographic Factors Associated With Smoking Among Malaysian Adults: A Cross‐Sectional Study [1]. It looks into how common smoking is among adults in Malaysia and discovers a number of sociodemographic variables that affect smoking behavior. By using a cross‐sectional approach, the study provides a brief summary of smoking behaviors and related traits in the general population.

The results offer important new information about the demographic elements that are associated with smoking prevalence, including age, gender, educational attainment, and socioeconomic status. For public health programs that attempt to lower smoking rates and customize interventions for certain high‐risk groups, this information is important.

Because it emphasizes the significance of raising knowledge about smoking and its negative health effects, this study has scientific significance that goes beyond Malaysia. The report emphasizes the necessity of successful and efficient public health initiatives that discuss both smoking and the rise of vaping as a substitute that many people believe to be safer. But is vaping safer or another health risk?

The results are especially pertinent in the Philippine setting. The nation has comparable problems with smoking and the growing vaping trend. There is a vaping shop on every corner. In their study, there is a prohibition of the sale of tobacco products to minors [1]. This is also in line with the Philippine context. The Republic Act No. 9211 stated that prohibition of selling tobacco products to those aged 18 years old and below. However, even near schools there are shops that sell tobacco and e‐cigarettes. Misconceptions regarding vaping's safety in comparison to traditional smoking are frequently caused by inaccurate information. Policymakers and health advocates in the Philippines can learn from this study how important it is to communicate clearly the dangers of smoking and vaping as well.

By comprehending the sociodemographic elements that impact smoking in Malaysia, comparable tactics to stop smoking and vaping might be created in the Philippine context. The research highlights the urgent need for evidence‐based public health strategies that can successfully address these problems and encourage adults to lead healthier lives.

## Author Contributions

**John Patrick C. Toledo:** conceptualization, writing – original draft, resources, writing – review and editing.

## Ethics Statement

Ethical standards are followed in the research.

## Conflicts of Interest

The author declares no conflicts of interest.

## Data Availability

The data that supports the findings of this study is available from the Health Sciences Reports, and other relevant materials.
